# Supplementary material for: Step Forward Cross Validation for Bioactivity Prediction: Out of Distribution Validation in Drug Discovery
Source: bioRxiv. 2024 Jul 4:2024.07.02.601740. Preprint. [Version 1] doi: 10.1101/2024.07.02.601740 (PMC11245006; doi:10.1101/2024.07.02.601740)
Supplement: Supplement 1 [file NIHPP2024.07.02.601740v1-supplement-1.pdf]

bioRxiv preprint doi: https://doi.org/10.1101/2024.07.02.601740; this version posted July 4, 2024. The copyright holder for this preprint (which was not certified by peer review) is the author/funder, who has granted bioRxiv a license to display the preprint in perpetuity. It is made available under aCC-BY 4.0 International license.

## Appendix 1

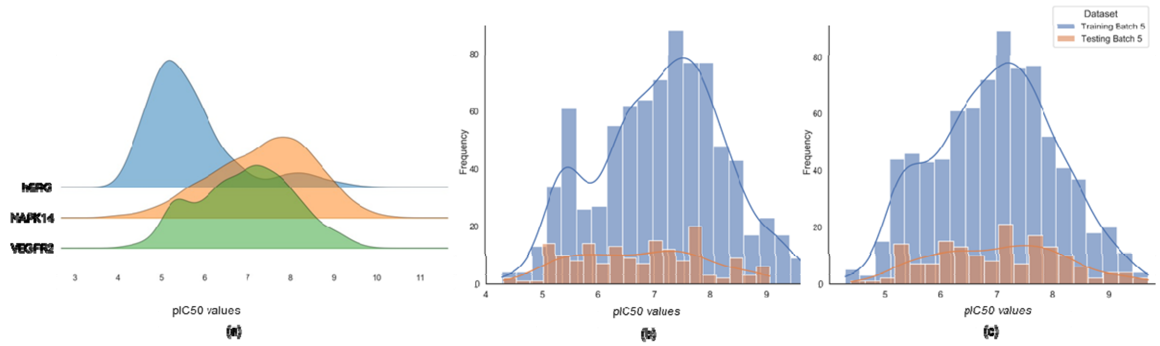


Figure S1: (a) Distribution of biological activity (pIC50 values) for the three protein target datasets. (b,c) Distribution of pIC50 values in the training set and test set of the 5th iteration for sorted SFCV (b) and unsorted SFCV (c). SFCV: Step-Forward Cross-Validation

bioRxiv preprint doi: https://doi.org/10.1101/2024.07.02.601740; this version posted July 4, 2024. The copyright holder for this preprint (which was not certified by peer review) is the author/funder, who has granted bioRxiv a license to display the preprint in perpetuity. It is made available under aCC-BY 4.0 International license.

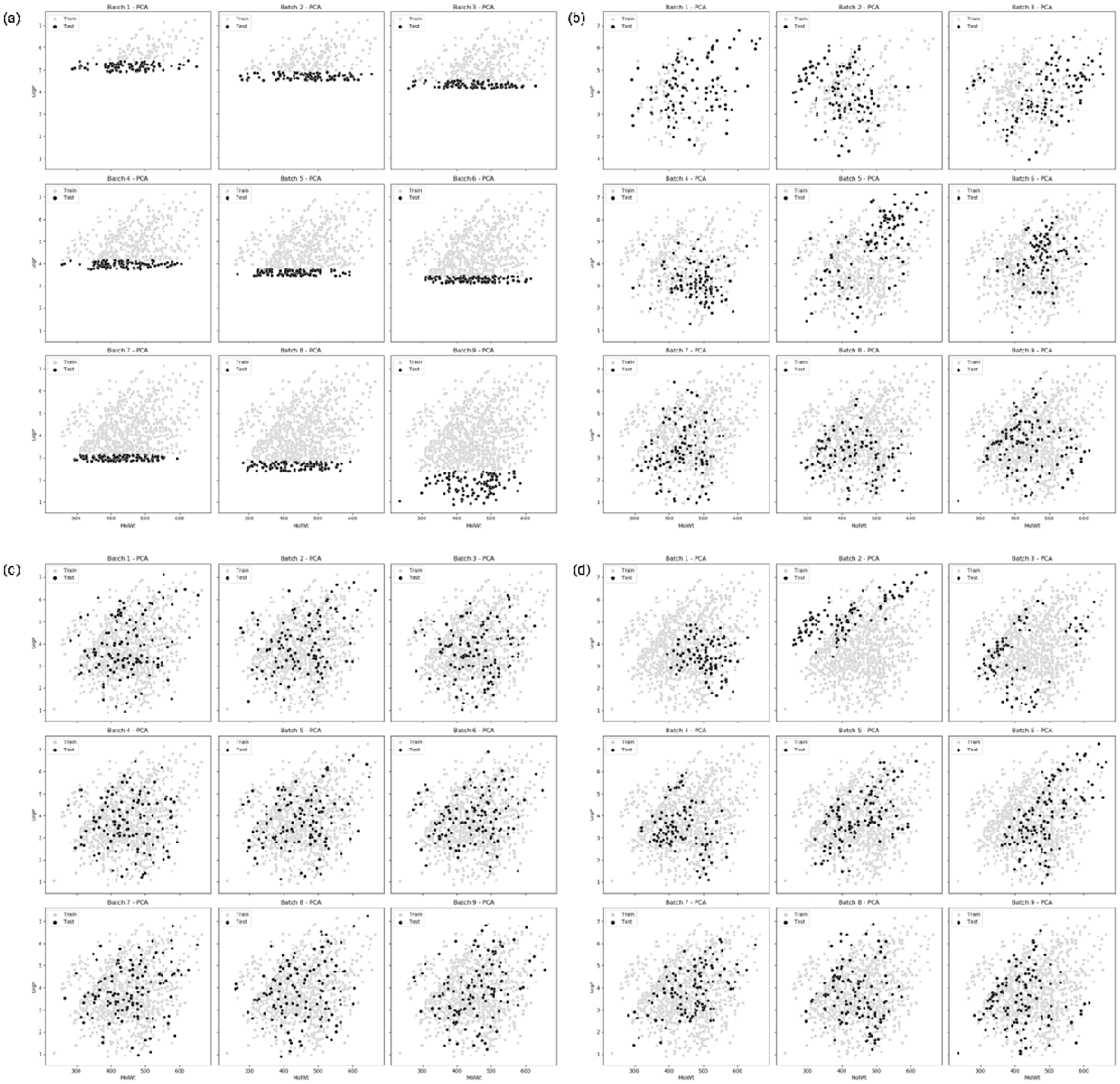


Figure S2: Comparison of logP and Molecular Weight for physico-chemical space for compounds selected as training and test sets across various iterations for the hERG target prediction task for (a) sorted SFCV, (b) unsorted SFCV, (c) cross-validation with random splits, and (d) cross-validation with scaffold splits for the (first) nine iterations for Random Forest models. SFCV: Step-Forward Cross-Validation

bioRxiv preprint doi: https://doi.org/10.1101/2024.07.02.601740; this version posted July 4, 2024. The copyright holder for this preprint (which was not certified by peer review) is the author/funder, who has granted bioRxiv a license to display the preprint in perpetuity. It is made available under aCC-BY 4.0 International license.

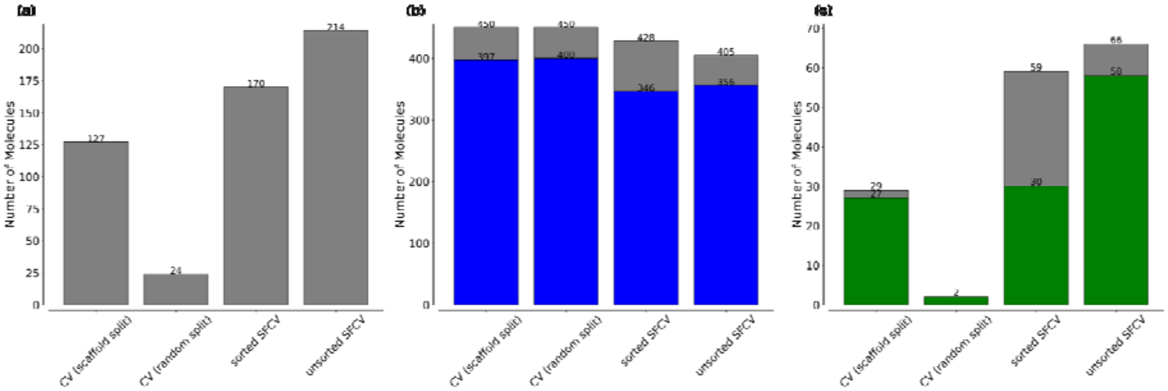


Figure S3: (a) The total number of compounds dissimilar to training data (Tc<0.55). (b) The total number of discovery compounds (pIC50<5.2) in the test set and the colored stacks show how many predictions are within a 0.5 log fold unit error range. (c) The number of discovery compounds dissimilar to training data (Tc<0.55) and the colored stacks show how many predictions are within a 0.5 log fold unit error range. Results refer to the hERG target prediction task across four validation methods: sorted SFCV, unsorted SFCV, cross-validation with random splits, and cross-validation with scaffold splits (combined for all test folds). Tc: Tanimoto Similarity; SFCV: Step-Forward Cross-Validation.

bioRxiv preprint doi: https://doi.org/10.1101/2024.07.02.601740; this version posted July 4, 2024. The copyright holder for this preprint (which was not certified by peer review) is the author/funder, who has granted bioRxiv a license to display the preprint in perpetuity. It is made available under aCC-BY 4.0 International license.

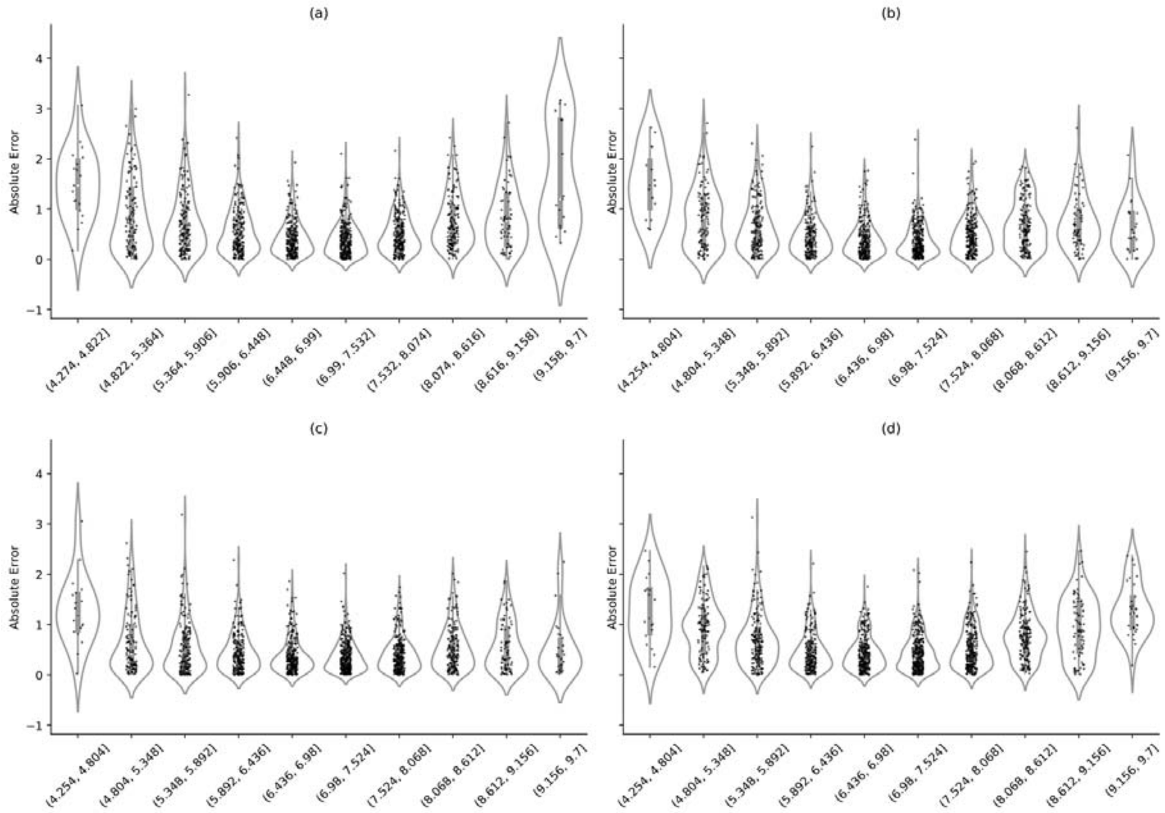


Figure S4: Absolute Error for VEGFR2 target prediction (sorted by pIC50 values) for (a) sorted SFCV, (b) unsorted SFCV, (c) cross-validation with random splits, and (d) cross-validation with scaffold splits. SFCV: Step-Forward Cross-Validation

bioRxiv preprint doi: https://doi.org/10.1101/2024.07.02.601740; this version posted July 4, 2024. The copyright holder for this preprint (which was not certified by peer review) is the author/funder, who has granted bioRxiv a license to display the preprint in perpetuity. It is made available under aCC-BY 4.0 International license.

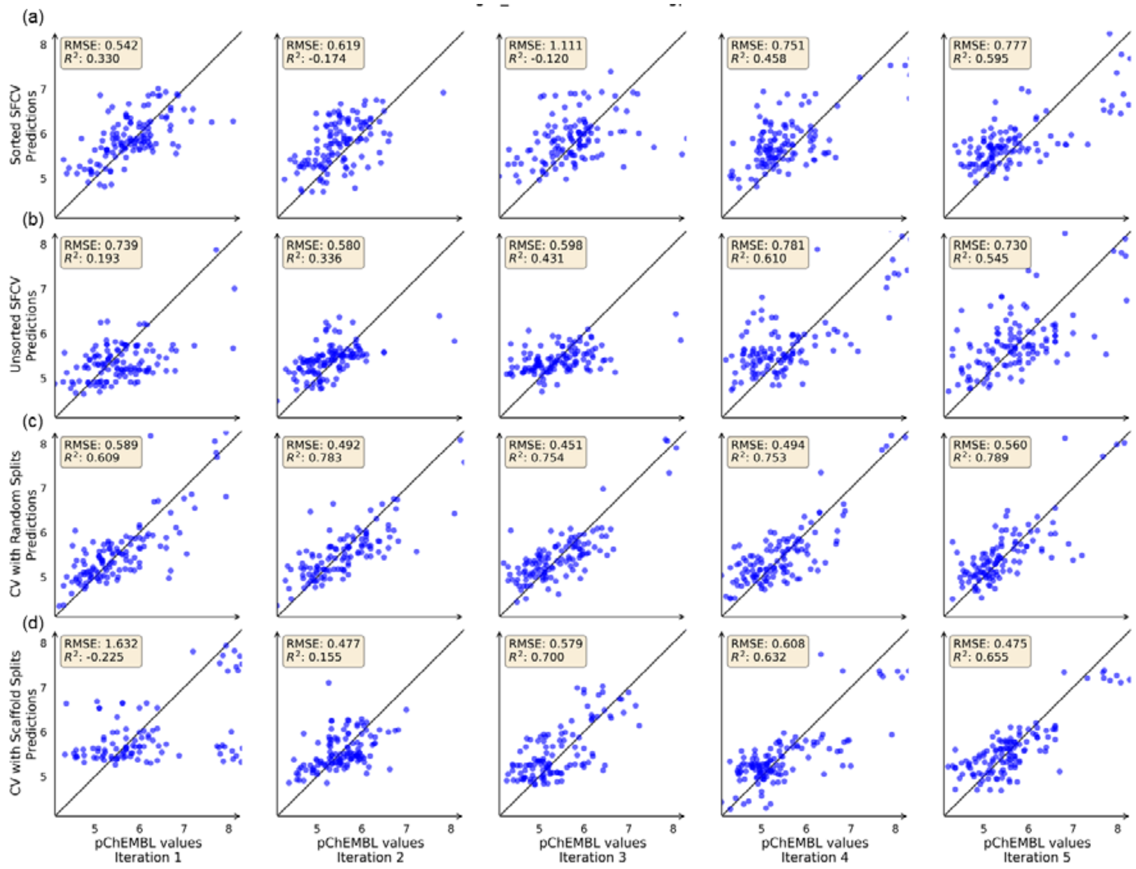


Figure S5: Parity plots for hERG target prediction for (a) sorted SFCV, (b) unsorted SFCV, (c) cross-validation with random splits, and (d) cross-validation with scaffold splits for the first five iterations for Random Forest models. SFCV: Step-Forward Cross-Validation

bioRxiv preprint doi: https://doi.org/10.1101/2024.07.02.601740; this version posted July 4, 2024. The copyright holder for this preprint (which was not certified by peer review) is the author/funder, who has granted bioRxiv a license to display the preprint in perpetuity. It is made available under aCC-BY 4.0 International license.

## Appendix 2

Table S1. Performance metrics for predicting the pIC50 for three protein targets in this work for each combination of models and validation techniques used in this study. Cross-validation (CV) is shown to perform better than other techniques.

| Target | Method | Validation | R2 (mean ± std) | RMSE (mean ± std) | Novelty Error (mean ± std) | Discovery Yield (mean ± std) |
|---|---|---|---|---|---|---|
| hERG | MLP | CV (Random) | **0.58 ± 0.10** | **0.66 ± 0.06** | 1.28 ± 0.34 | **0.70 ± 0.11** |
| hERG | MLP | CV (Scaffold) | 0.08 ± 0.58 | 0.81 ± 0.21 | 1.07 ± 0.59 | 0.57 ± 0.07 |
| hERG | MLP | SFCV (Sorted) | 0.07 ± 0.65 | 0.85 ± 0.25 | 0.99 ± 0.37 | 0.61 ± 0.11 |
| hERG | MLP | SFCV (Unsorted) | 0.26 ± 0.47 | 0.79 ± 0.16 | **0.80 ± 0.31** | 0.64 ± 0.10 |
| hERG | RF | CV (Random) | **0.70 ± 0.07** | **0.55 ± 0.07** | 0.74 ± 0.51 | **0.74 ± 0.06** |
| hERG | RF | CV (Scaffold) | 0.41 ± 0.31 | 0.69 ± 0.35 | 0.82 ± 0.80 | 0.59 ± 0.14 |
| hERG | RF | SFCV (Sorted) | 0.32 ± 0.46 | 0.73 ± 0.20 | 0.87 ± 0.27 | 0.47 ± 0.12 |
| hERG | RF | SFCV (Unsorted) | 0.52 ± 0.18 | 0.66 ± 0.09 | **0.65 ± 0.20** | 0.62 ± 0.11 |
| hERG | xgboost | CV (Random) | **0.70 ± 0.09** | **0.55 ± 0.07** | 0.72 ± 0.49 | **0.71 ± 0.10** |
| hERG | xgboost | CV (Scaffold) | 0.38 ± 0.27 | 0.71 ± 0.33 | 0.80 ± 0.82 | 0.58 ± 0.10 |
| hERG | xgboost | SFCV (Sorted) | 0.35 ± 0.43 | 0.71 ± 0.18 | 0.84 ± 0.32 | 0.45 ± 0.14 |
| hERG | xgboost | SFCV (Unsorted) | 0.51 ± 0.22 | 0.66 ± 0.09 | **0.60 ± 0.25** | 0.63 ± 0.11 |
| MAPK14 | MLP | CV (Random) | **0.66 ± 0.07** | **0.67 ± 0.05** | **0.67 ± 0.66** | **0.65 ± 0.05** |
| MAPK14 | MLP | CV (Scaffold) | 0.39 ± 0.41 | 0.83 ± 0.12 | 1.04 ± 0.45 | 0.54 ± 0.07 |
| MAPK14 | MLP | SFCV (Sorted) | 0.39 ± 0.21 | 0.88 ± 0.17 | 1.05 ± 0.40 | 0.47 ± 0.07 |
| MAPK14 | MLP | SFCV (Unsorted) | 0.56 ± 0.18 | 0.76 ± 0.14 | 0.91 ± 0.55 | 0.59 ± 0.11 |

bioRxiv preprint doi: https://doi.org/10.1101/2024.07.02.601740; this version posted July 4, 2024. The copyright holder for this preprint (which was not certified by peer review) is the author/funder, who has granted bioRxiv a license to display the preprint in perpetuity. It is made available under aCC-BY 4.0 International license.

| | | | | | | |
|---|---|---|---|---|---|---|
| MAPK14 | RF | CV (Random) | **0.76 ± 0.04** | **0.57 ± 0.05** | **0.76 ± 0.39** | **0.74 ± 0.04** |
| MAPK14 | RF | CV (Scaffold) | 0.62 ± 0.18 | 0.67 ± 0.08 | 0.88 ± 0.22 | 0.66 ± 0.08 |
| MAPK14 | RF | SFCV (Sorted) | 0.54 ± 0.09 | 0.77 ± 0.11 | 0.93 ± 0.17 | 0.63 ± 0.05 |
| MAPK14 | RF | SFCV (Unsorted) | 0.67 ± 0.09 | 0.67 ± 0.10 | 0.93 ± 0.37 | 0.69 ± 0.06 |
| MAPK14 | xgboost | CV (Random) | **0.74 ± 0.05** | **0.59 ± 0.06** | **0.79 ± 0.50** | **0.73 ± 0.04** |
| MAPK14 | xgboost | CV (Scaffold) | 0.53 ± 0.26 | 0.73 ± 0.10 | 0.95 ± 0.13 | 0.60 ± 0.08 |
| MAPK14 | xgboost | SFCV (Sorted) | 0.48 ± 0.12 | 0.81 ± 0.12 | 0.98 ± 0.19 | 0.61 ± 0.06 |
| MAPK14 | xgboost | SFCV (Unsorted) | 0.64 ± 0.09 | 0.70 ± 0.09 | 1.03 ± 0.34 | 0.67 ± 0.08 |
| VEGFR2 | MLP | CV (Random) | **0.53 ± 0.06** | **0.74 ± 0.06** | 1.04 ± 0.59 | **0.59 ± 0.03** |
| VEGFR2 | MLP | CV (Scaffold) | 0.27 ± 0.21 | 0.89 ± 0.11 | **0.87 ± 0.34** | 0.44 ± 0.08 |
| VEGFR2 | MLP | SFCV (Sorted) | 0.04 ± 0.35 | 1.02 ± 0.13 | 1.33 ± 0.17 | 0.39 ± 0.06 |
| VEGFR2 | MLP | SFCV (Unsorted) | 0.43 ± 0.16 | 0.81 ± 0.10 | 0.99 ± 0.37 | 0.48 ± 0.09 |
| VEGFR2 | RF | CV (Random) | **0.66 ± 0.04** | **0.63 ± 0.05** | 0.91 ± 0.35 | **0.67 ± 0.05** |
| VEGFR2 | RF | CV (Scaffold) | 0.46 ± 0.19 | 0.77 ± 0.08 | **0.71 ± 0.23** | 0.50 ± 0.10 |
| VEGFR2 | RF | SFCV (Sorted) | 0.43 ± 0.13 | 0.80 ± 0.07 | 0.84 ± 0.18 | 0.55 ± 0.06 |
| VEGFR2 | RF | SFCV (Unsorted) | 0.58 ± 0.08 | 0.70 ± 0.06 | 0.86 ± 0.28 | 0.57 ± 0.07 |
| VEGFR2 | xgboost | CV (Random) | **0.62 ± 0.03** | **0.67 ± 0.04** | 0.95 ± 0.28 | **0.59 ± 0.07** |
| VEGFR2 | xgboost | CV (Scaffold) | 0.41 ± 0.26 | 0.79 ± 0.10 | **0.71 ± 0.21** | 0.47 ± 0.11 |
| VEGFR2 | xgboost | SFCV (Sorted) | 0.41 ± 0.12 | 0.82 ± 0.07 | 0.88 ± 0.17 | 0.55 ± 0.06 |
| VEGFR2 | xgboost | SFCV (Unsorted) | 0.54 ± 0.07 | 0.74 ± 0.04 | 0.86 ± 0.19 | 0.55 ± 0.05 |
